# A Rare *ABCB5* Variant in a Familial Case of Intrahepatic Cholestasis of Pregnancy: A Potential Novel Genetic Contributor

**DOI:** 10.3390/jcm14165618

**Published:** 2025-08-08

**Authors:** Małgorzata Kędzia, Ewa Wender-Ożegowska, Justyna Dąbrowska, Paweł P. Jagodziński, Adrianna Mostowska

**Affiliations:** 1 Department of Reproduction, Chair of Reproduction and Perinatal Medicine, Poznan University of Medical Sciences, 60-535 Poznan, Poland; mszczepanska@ump.edu.pl (M.K.); ewozegow@ump.edu.pl (E.W.-O.); 2 Department of Biochemistry and Molecular Biology, Poznan University of Medical Sciences, 60-781 Poznan, Poland; jdabrowska@ump.edu.pl (J.D.); pjagodzi@ump.edu.pl (P.P.J.)

**Keywords:** intrahepatic cholestasis of pregnancy, WES, ABCB5

## Abstract

**Background/Objectives:** Intrahepatic cholestasis of pregnancy (ICP) is a pregnancy-specific liver disorder with a multifactorial pathogenesis and a well-established genetic component. While pathogenic variants in genes such as *ABCB4* and *ABCB11* are implicated in a subset of cases, many remain genetically unexplained. This study aimed to investigate the genetic background of ICP in a multi-generational family with recurrent hepatobiliary disease. **Methods:** Whole-exome sequencing was performed on the proband and five female relatives. Variant filtering prioritized rare, exonic or splice-site variants predicted to undergo damage by *in silico* tools and which were present in all affected family members. Identified variants were assessed using population databases and compared with a control group of 433 unrelated women with uncomplicated pregnancies. Variant confirmation was performed using Sanger sequencing and high-resolution melting analysis. **Results:** No pathogenic variants were identified in known ICP-associated genes. However, a rare heterozygous missense variant in *ABCB5* (c.1610G>A; p.Arg537His; rs779950110) was found in all affected individuals and two younger female relatives. This variant is exceedingly rare in population databases, absent in controls, and predicted to be pathogenic by multiple algorithms. *ABCB5*, although not previously linked to ICP, is an ATP-binding cassette transporter expressed in various tissues, including liver compartments. **Conclusions:** This study reports a novel *ABCB5* variant segregating with ICP and early-onset hepatobiliary disease in a family. These findings suggest *ABCB5* as a potential new susceptibility gene in ICP, warranting further functional investigation.

## 1. Introduction

Intrahepatic cholestasis of pregnancy (ICP), unlike preeclampsia, HELLP syndrome, or acute fatty liver of pregnancy, does not pose an immediate threat to maternal life. Nevertheless, it warrants close clinical attention due to its association with an increased risk of adverse perinatal outcomes, including preterm delivery, meconium-stained amniotic fluid, and intrauterine fetal death (IUFD) [1]. This pregnancy-specific liver disorder affects approximately 0.5–2% of pregnant women, with geographic and ethnic variations in its prevalence [2].

The diagnosis of ICP is based on elevated total serum bile acid (TSBA) levels and abnormal liver function tests. A TSBA concentration of ≥40 µmol/L indicates moderate disease severity, while levels ≥100 µmol/L are strongly associated with an increased risk of IUFD [3,4].

Despite treatment and reductions in serum bile acid levels, adverse pregnancy outcomes may still occur in ICP. Neonatal complications can arise unpredictably and often without preceding clinical signs or symptoms, posing a significant challenge for timely intervention and risk stratification [5]. This unpredictability underscores the need for improved biomarkers and a deeper understanding of the underlying pathophysiology.

The pathogenesis of ICP is multifactorial, involving hormonal, environmental, and genetic factors [6,7]. Notably, elevated levels of estrogen and progesterone metabolites are thought to impair hepatocellular bile acid transporters. The accumulation of progesterone metabolites, particularly in women receiving progesterone supplementation, may explain this subgroup’s heightened risk of ICP.

The higher incidence of ICP in certain ethnic populations, its familial clustering, earlier onset in subsequent pregnancies, and frequent manifestation during a woman’s first pregnancy all strongly suggest a genetic predisposition [8]. Molecular studies have identified pathogenic variants in genes encoding bile acid transport proteins, such as *ABCB4* (OMIM *171060) and *ABCB11* (OMIM *603201), which may predispose individuals to ICP, particularly in early-onset and severe cases [9,10,11]. These genes encode the phosphatidyl choline floppase and the bile salt export pump, respectively. Rare heterozygous variants in *ABCB4* and *ABCB11* have been shown to account for up to 25% of severe, early-onset ICP cases [12].

Other genes potentially implicated in bile acid homeostasis include *ATP8B1* (OMIM *602397), *NR1H4* (OMIM *603826), and *TJP2* (OMIM *607709), though the clinical relevance of their variants in the general population remains uncertain [7,11,13,14,15]. Moreover, a recent genome-wide association study (GWAS) meta-analysis has suggested that common variants in liver-enriched genes and/or liver cis-regulatory elements may also contribute to ICP susceptibility, highlighting a polygenic component to the disease’s genetic architecture [2].

In recent years, the application of next-generation sequencing techniques has garnered increasing interest in identifying rare pathogenic variants in atypical or severe cases of ICP. Familial clustering and early onset are particularly suggestive of an underlying genetic component. However, comparative genomic data from healthy pregnant women without a history of ICP who deliver at term remain scarce, limiting the interpretation of genetic findings in affected individuals [16,17,18].

This study aimed to analyze a clinical case of a pregnant woman with early-onset ICP and a family history of the condition using whole-exome sequencing (WES). The genomic findings from the proband and her affected relatives were compared with those of a control group of healthy pregnant women without a history of cholestasis who delivered healthy, full-term neonates.

The overarching objective was to identify potential genetic variants that may confer susceptibility to ICP and to contribute to a deeper understanding of its genetic background.

## 2. Materials and Methods

### 2.1. Case Presentation and Control Group

The proband was admitted to the Gynecological and Obstetric Hospital of the Medical University of Poznan (Poland) at 24 weeks of gestation, presenting with pruritus, primarily affecting the palms and soles, and worsening at night. The itching had been present for several weeks. After obtaining informed consent, she underwent standardized clinical evaluations and was diagnosed with severe intrahepatic cholestasis of pregnancy. This diagnosis was established based on the presence of unexplained pruritus accompanied by elevated bile acid levels (≥18 µmol/L) and liver dysfunction after excluding other liver diseases, including viral hepatitis, acute fatty liver of pregnancy, preeclampsia, primary biliary cirrhosis, and HELLP syndrome [19]. A detailed family history of the proband was gathered.

Peripheral blood samples for genetic testing were collected from the proband and five available female relatives, three of whom also had ICP. Additionally, blood samples were obtained from 433 unrelated healthy women with uncomplicated pregnancies, all of whom delivered in 2020–2022 at the same hospital (median age 32; range: 19–39). None of the women in the control group had a history of cholestasis in any previous pregnancies or experienced pruritus in their current or past pregnancies. All study participants, including members of the tested family and the control group, were Caucasians of Polish origin. The Polish population is considered genetically homogeneous, with only minor regional variation reported [20]. DNA was extracted from peripheral blood leucocytes using the standard salt-out method. The study design and experimental protocols were approved by the Institutional Review Board of Poznan University of Medical Sciences, Poland (approval no 1062/16; approval date: 1 December 2016). Written informed consent was obtained from all participants.

### 2.2. Whole-Exome Sequencing (WES)

WES was performed on the proband and her five female relatives. Library preparation for WES was carried out using the TruSeq DNA Exome kit (Illumina Inc., San Diego, CA, USA). Sequencing was conducted on an Illumina platform with paired-end index sequencing (2 × 100 bp). Raw sequencing data were processed following a previously described pipeline [21] and aligned to the GRCh38/hg38 reference genome. Identified variants were annotated with functional information, *in silico* pathogenicity predictions, population frequency, and clinical significance based on ClinVar (https://www.ncbi.nlm.nih.gov/clinvar/, accessed on 2 June 2025) and the Human Gene Mutation Database (HGMD, http://www.hgmd.cf.ac.uk, accessed on 2 June 2025). Predicted variant effects were assessed using multiple *in silico* tools. Individual predictions and meta-scores were retrieved from the VarSome genomic variant search engine (https://varsome.com/, accessed on 2 June 2025) and the Ensemble genome browser (https://www.ensembl.org/index.html, accessed on 2 June 2005). Additionally, the Combined Annotation Dependent Depletion (CADD) and Adaptive Boosting (ADA) scores were analyzed for missense variants and those with potential splicing effects.

### 2.3. Variant Filtering and Prioritizing

To reduce the list of candidate variants associated with ICP, only exonic and splicing alterations (excluding synonymous variants) with a minor allele frequency <0.001 in the gnomAD European (non-Finnish; https://gnomad.broadinstitute.org/, accessed on 2 June 2025) population were considered. An exception was made for variants in genes previously linked to ICP, including, among others, *ABCB4*, *ABCB11*, and *ATP8B1*, all of which were utilized in the analysis. Likely gene-disrupting variants were defined as stop-gain, stop-loss, frameshift, and splicing variants with an ADA score ≥ 0.9, as well as missense variants with a CADD score > 20, provided that they were predicted to be pathogenic or damaging by at least half of the *in silico* prediction algorithms. Variants were prioritized if they were identified in genes previously associated with ICP risk or genes involved in bile acid synthesis or transport. Those meeting stringent selection criteria were classified as pathogenic or likely pathogenic, depending on their annotation in the ClinVar or HGMD databases. Additionally, to be considered disease-associated, a specific variant had to be present in all affected family members with ICP.

### 2.4. Protein Structure Impact and Cross-Species Conservation Analysis

The structural location and potential functional impact of the ABCB5 p.Arg537His (rs779950110) variant identified in this study were analyzed using the AlphaFold Protein Structure Database (https://alphafold.ebi.ac.uk/, accessed on 18 July 2025), which provides open-access predictions for over 200 million proteins [22]. The AlphaMissense score and predicted Local Distance Difference Test (pLDDT) confidence scores were obtained from the AlphaFold-integrated annotations available for ABCB5.

To assess evolutionary conservation, multiple sequence alignments of ABCB5 orthologs across eight vertebrate species were performed using data from the UCSC Genome Browser (version 114) (https://genome-euro.ucsc.edu/, accessed on 18 July 2025).

### 2.5. Confirmation Analyses

The likely pathogenic *ABCB5* variant (rs779950110) was validated using Sanger sequencing. Sequencing was performed with the BigDye™ Terminator v3.1 Ready Reaction Cycle Sequencing Kit on an ABI3730 Genetic Analyzer (Applied Biosystems, Foster City, CA, USA). The primers used for sequencing were F: ATCACGCTGCCTTCTGTTTC and R: GCAAACCACTTGGAATGGAG (PCR product size: 489 bp). To assess the presence of the rs779950110 variant in the control group, a high-resolution melting curve (HRM) analysis was employed. DNA from the proband, her family members, and healthy controls was amplified using the following primers: F—ATGAGTGGAGGGCAGAAACA and R—TTTCTGAATCCAGGGCAGAC (PCR product size: 103 bp). The amplified fragments were then subjected to HRM analysis with temperature increments of 0.1 °C, ranging from 78 °C to 93 °C. HRM analyses were conducted using the LightCycler 96 system (Roche Diagnostics, Mannheim, Germany).

## 3. Results

### 3.1. Clinical Findings

**Figure 1:**
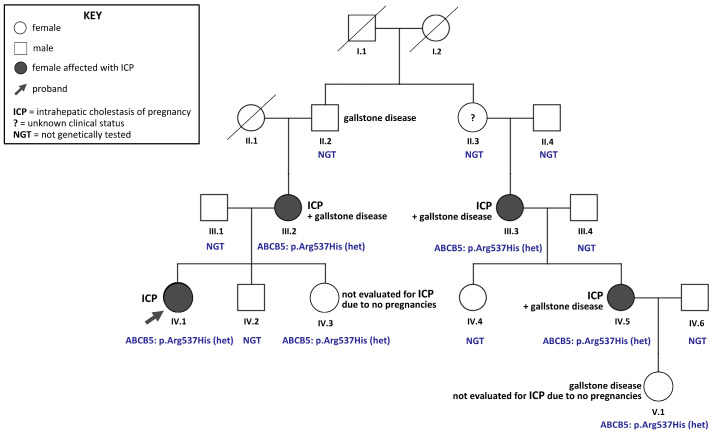
Pedigree of the proband’s family, indicating the individuals affected by intrahepatic cholestasis of pregnancy (ICP) and gallstone disease. The proband (IV.1) is indicated by an arrow. Circles represent females, and squares represent males. Filled symbols denote individuals affected by ICP. Family members who underwent genetic testing are marked; all tested individuals (III.2, III.3, IV.1, IV.3, IV.5, and V.1) were found to carry a likely pathogenic variant of the *ABCB5* gene (rs779950110) in the heterozygous state. This c.1610G>A transition in exon 14 results in an arginine-to-histidine substitution at position 537 of the encoded protein (p.Arg537His).

A 33-year-old woman (IV.1, Figure 1) was admitted to the hospital at 24 weeks of gestation due to pruritus, predominantly affecting the palms and soles and worsening at night. The itching had been present for several weeks. This was her second pregnancy; the first had ended in a spontaneous miscarriage after 12 weeks, two years earlier. Her medical history was notable for cerebral palsy. Her child was born eight weeks prematurely, with a birth weight of 1760 g, following the preterm premature rupture of membranes. No other comorbidities, allergies, or liver disorders were reported.

Initial laboratory tests upon admission revealed markedly elevated total bile acids (TBA, 171.8 µmol/L), liver enzymes (ALT 867.1 U/L; AST 492.8 U/L), and bilirubin (1.54 mg/dL). Serological testing excluded active liver disease. Further investigations, including screening for hemochromatosis, Wilson’s disease, and autoimmune liver diseases, were all negative. Abdominal ultrasound showed no abnormalities in the liver, bile ducts, or gallbladder. Blood morphology parameters, including hemoglobin and platelet counts, were within normal limits.

Fetal ultrasound revealed that normal development, amniotic fluid volume, umbilical artery Doppler flow, and cardiotocography findings were also normal.

A diagnosis of severe ICP was established, and treatment with ursodeoxycholic acid at a dose of 1500 mg daily was initiated. Although bile acid levels initially declined, they subsequently rose again to 128.0 µmol/L. The patient received dexamethasone for fetal lung maturation. Following consultation with a gastroenterologist, additional therapy with cholestyramine and ornithine aspartate was initiated.

Three days after the introduction of combined therapy, the patient’s platelet count decreased to 110.0 G/L and hemoglobin decreased to 5.4 mmol/L. Methylprednisolone was added to her treatment regimen, resulting in the normalization of these parameters within 7 days. Coagulation profiles remained within normal ranges throughout hospitalization, and vitamin K supplementation was not required.

**Figure 2:**
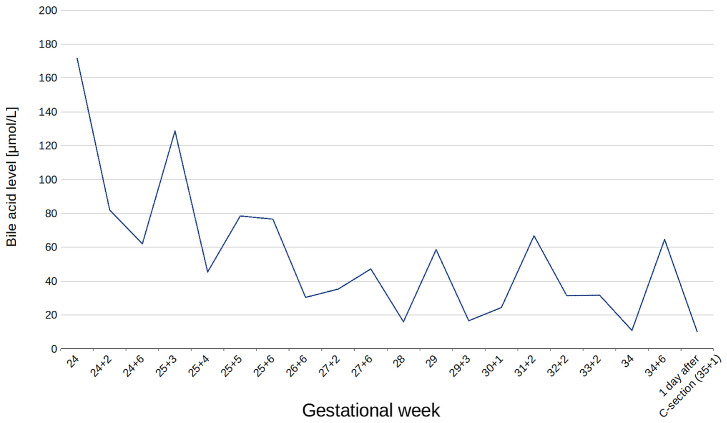
Changes in total serum bile acid levels in the proband between the 24th and 35th week of gestation. A transient decrease in bile acids was observed following the initiation of ursodeoxycholic acid monotherapy, followed by fluctuating levels during subsequent combination treatment.

**Figure 3:**
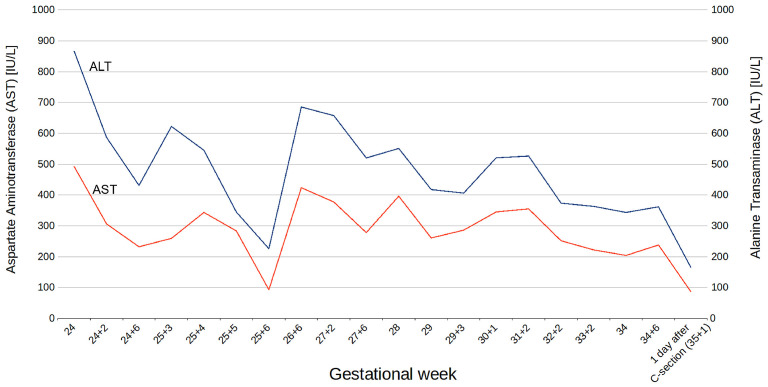
Changes in serum aminotransferase levels (ALT and AST) in the proband between the 24th and 35th week of gestation. A gradual decline in transaminase levels was observed following the initiation of treatment.

Bile acid levels fluctuated during hospitalization, ranging from mild to moderate cholestasis (lowest level at 10.0 µmol/L at 34 weeks, increasing to 64.6 µmol/L for the following week; Figure 2). Changes in aminotransferase levels from the 24th to the 35th week of pregnancy are shown in Figure 3.

At 35 weeks, due to the recurrent elevation of bile acid (above 60.0 µmol/L), persistent symptoms, and uterine contractions, a cesarean section was performed. A healthy male infant weighing 2550 g was delivered, with Apgar scores of 10 at 1 and 5 min. By the third postpartum day, the patient’s bile acid levels had decreased to 10.0 µmol/L, with ALT at 64.5 U/L and AST at 86 U/L.

Family history revealed a pattern suggestive of hereditary cholestasis (Figure 1). The proband’s maternal grandfather (II.2) had gallstone disease. Her mother (III.2) experienced five pregnancies, including two miscarriages and three live births, two of which were preterm (approximately eight weeks early). She reported intense pruritus beginning in the fifth month of each successful pregnancy, which resolved postpartum. She also experienced itching while taking oral contraceptives and discontinued them after about 10 days. At age 40, she underwent a cholecystectomy due to gallstones.

The proband’s younger sister (IV.3) has not been pregnant and has no history of gallstone disease. On the maternal side, the proband’s aunt (III.3) experienced ICP in both her pregnancies and also underwent cholecystectomy. Among her daughters, one (IV.4) had no pregnancy complications, while the other (IV.5) experienced ICP during both pregnancies, pruritus with oral contraceptives, and required cholecystectomy. The daughter of IV.5 (V.1) also underwent cholecystectomy at the age of 11.

### 3.2. Molecular Analyses

**Figure 4:**
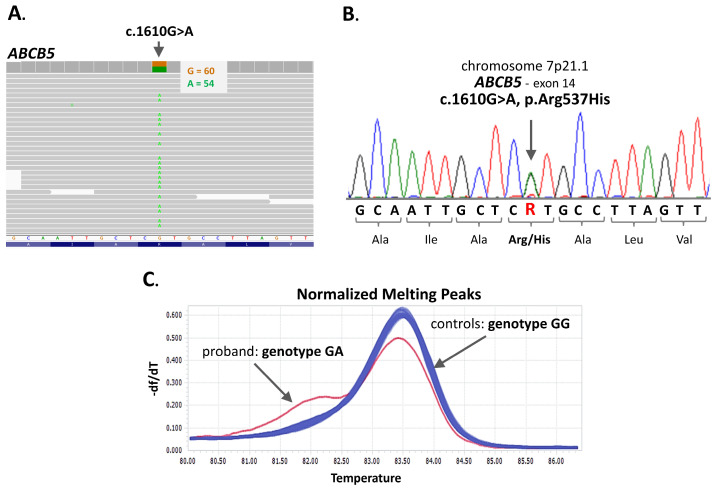
The detection of an *ABCB5* missense variant. In the proband with intrahepatic cholestasis of pregnancy, whole-exome sequencing identified a likely pathogenic variant c.1610G>A in exon 14 of the *ABCB5* gene (**A**). The presence of this heterozygous transition, resulting in a p.Arg537His substitution, was confirmed by Sanger sequencing (**B**) and high-resolution melting (HRM) curve analysis (**C**). The HRM derivative plot shows a distinct melting profile corresponding to the heterozygous genotype, differentiating it from the wild-type control.

Following variant filtering, prioritization, and validation, a likely pathogenic variant in the *ABCB5* gene (OMIM *611785) was identified (Figure 4). This known single-nucleotide transition, c.1610G>A (rs779950110), located in exon 14, results in an arginine-to-histidine substitution at codon 537 (p.Arg537His). The frequency of this variant in the gnomAD European (non-Finnish) population is 0.0000171, while in all gnomAD exome individuals, the frequency of the A allele is 0.0000349 (gnomAD Exomes, version: 4.1; Supplementary Figure S1). The p.Arg537His variant, with a CADD score of 28.4, is classified as pathogenic (strong, moderate, or supporting) by the majority of individuals using *in silico* prediction tools, including MutPred, LIST-S2, Mutation assessor, EIGEN, EIGEN PC, MVP, Polyphen, PROVEAN, SIFT, and SIFT4G. Additionally, several meta-predictors, such as MetaRNN, MetaLR, MetaSVM, and REVEL, also support its pathogenicity (Supplementary Table S1). This *ABCB5* variant was detected in heterozygous form in the proband and five of her female relatives who were available for testing (Figure 1, Supplementary Figures S2 and S3). Among them, three had a history of ICP and gallstone disease, while the remaining two were not evaluated for ICP due to their young age and absence of pregnancy. The presence of the p.Arg537His variant in all six tested family members was confirmed by standard Sanger sequencing and HRM (Supplementary Figure S3). In contrast, the HRM analysis of DNA samples from the control group (n = 433) did not detect this substitution.

No other pathogenic or likely pathogenic variants shared by all affected family members with ICP were identified. We specifically analyzed sequencing data for the three major genes most commonly implicated in intrahepatic cholestasis of pregnancy: *ABCB4*, *ABCB11*, and *ATP8B1*. In *ABCB4*, a single deep intronic variant (rs10647775) was detected in the proband; however, it was not predicted to affect splicing or gene function. In *ABCB11*, one common synonymous variant (rs497692, p.Ala1028Ala), three variants located in the region encoding 3′UTR (rs496550, rs495714, and rs473351), and two intronic variants (rs579275 and rs853789) were identified. None of these variants has been previously associated with ICP or considered functionally significant. In *ATP8B1*, which is implicated in progressive familial intrahepatic cholestasis, six intronic variants were identified (rs4306606, rs9964678, rs317838, rs35718397, rs319439, and rs4940989), as well as one missense variant (rs150860808, c.913T>A, p.Ple305Ile). This missense variant, with a low CADD score of 5.839, was present in the heterozygous state only in the proband and her sister.

### 3.3. In Silico Structural and Evolutionary Conservation Analysis

**Figure 5:**
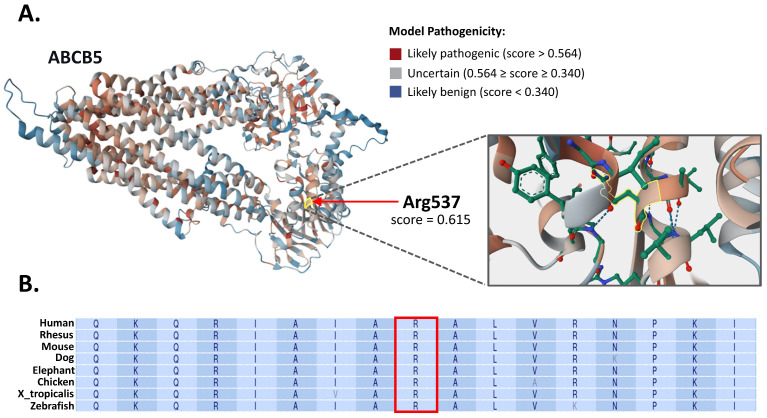
The predicted structural impact of ABCB5 p.Arg537His (rs779950110) and cross-species conservation. (**A**) The AlphaFold-predicted structure of ABCB5 (UniProt ID Q2M3G0) was retrieved from the AlphaFold Protein Structure Database (https://alphafold.ebi.ac.uk/, accessed on 18 July 2025). The structure is color-coded according to the average AlphaMissense pathogenicity score. The side chain of residue p.Arg537 is highlighted in yellow. The AlphaMissense score at this position is 0.615, indicating a high likelihood of structural disruption. The predicted Local Distance Difference Test confidence score for p.Arg537 is 94.35, indicating high local model confidence and near-experimental accuracy (estimated error of ~1 Å). (**B**) Multiple sequence alignment of the ABCB5 protein across eight vertebrate species (data retrieved from the UCSC Genome Browser 114, https://genome-euro.ucsc.edu/, accessed on 18 July 2025) demonstrates strong evolutionary conservation of the arginine residue at position 537 (highlighted within the frame).

Structural analysis of the ABCB5 p.Arg537His variant revealed a high likelihood of potential disruption to the protein structure (Figure 5A). The AlphaMissense score at position Arg537 was 0.615, exceeding the threshold of 0.564 typically associated with likely pathogenic substitutions. Furthermore, the pLDDT confidence score for this residue was 94.35, indicating very high local model confidence. Scores above 90 correspond to near-experimental accuracy, with an estimated positional error of approximately 1 Å [22].

The p.Arg537His substitution is located within the N-terminal “ABC transporter 1” domain of ABCB5 (UniProt Q2M3G0; amino acids 386–622), which corresponds to nucleotide-binding site 1 (NBS1) in canonical ABC transporter architecture. Sequence analysis indicates that this NBS1 is degenerate, as it lacks the conserved Walker A motif necessary for ATP binding and hydrolysis, rendering it catalytically inactive [23,24].

In addition, the multiple sequence alignment of ABCB5 orthologs across eight vertebrate species demonstrated strong evolutionary conservation of the arginine residue at position 537 (Figure 5B), supporting its potential structural and functional importance.

## 4. Discussion

In the present study, we describe and molecularly characterize a familial case of ICP, which is the most common pregnancy-specific liver disorder characterized by pruritus and impaired bile acid homeostasis. Approximately 15–35% of affected women reported a positive family history, most commonly involving first-degree relatives such as mothers or sisters [25,26]. A nationwide Danish cohort study involving 668,461 primiparous women demonstrated a significantly increased risk of ICP among co-twins (~5-fold) and the first-degree relatives (~2.5-fold) of affected individuals [27]. Similarly, a population-based study from Finland revealed that mothers, sisters, and daughters of ICP patients have a significantly elevated risk of hepatic dysfunction during pregnancy [28]. Taken together, these findings, along with the consistent familial clustering of ICP cases across generations, confirm a substantial genetic contribution to ICP pathogenesis and suggest an autosomal dominant inheritance pattern with incomplete penetrance [28]. This mode of inheritance may account for the observed variable expressivity and the presence of asymptomatic carriers within affected families, posing challenges for both diagnosis and genetic counseling.

Consistent with this proposed inheritance model, the pedigree in our study demonstrates a clear familial aggregation of hepatobiliary disease (Figure 1). The proband’s mother (III.2) experienced severe ICP and subsequently underwent cholecystectomy at the age of 40 for gallstone disease. Additional maternal relatives further supported this hereditary pattern: the proband’s aunt (III.3) had ICP in both pregnancies and underwent cholecystectomy, and her daughter (IV.5) experienced ICP in both pregnancies, developed pruritus in response to oral contraceptives, and also required cholecystectomy. Notably, the cousin’s (IV.5) daughter (V.1) underwent cholecystectomy at the age of 11, highlighting a possible transgenerational expression of hepatobiliary disease even in the absence of pregnancy. Interestingly, the proband’s maternal grandfather (II.2) also had gallstone disease. Although male carriers of pathogenic variants are typically asymptomatic for cholestasis due to the pregnancy-specific nature of ICP, previous studies suggest that they may manifest subclinical or alternate hepatobiliary phenotypes, such as early or recurrent gallstone formation, particularly in the presence of *ABCB4* mutations [29,30]. Unfortunately, the grandfather’s DNA sample was unavailable for molecular testing, limiting our ability to evaluate the complete segregation of the identified variant across all affected family members.

WES was performed on the proband and five female relatives to investigate the genetic basis of ICP in this family. No common or rare pathogenic or likely pathogenic variants were identified in the major genes previously implicated in ICP, including, among others, *ABCB4*, *ABCB11*, and *ATP8B1*. However, the contribution of variants in these genes cannot be entirely excluded, as disease-associated nucleotide changes may reside outside the coding regions.

Although WES is a valuable tool for detecting variants in protein-coding regions, it has important limitations [31]. It does not include most non-coding regions of the genome, including regulatory elements, deep intronic sequences, or structural changes, all of which could contribute to disease. In addition, coverage across exons can vary, and some regions, such as those rich in GC content or highly repetitive, may be poorly covered or misrepresented. WES also has limited the ability to detect structural variants, larger insertions or deletions, and copy number changes [32]. For these reasons, we recognize that some potentially relevant variants in our proband may not have been identified. To overcome these limitations, whole-genome sequencing would be required to allow for a more comprehensive search, including coding and non-coding variants and complex structural alterations [33].

The only variant meeting stringent pathogenicity selection criteria was a rare heterozygous missense change in the *ABCB5* gene. This c.1610G>A transition (rs779950110) results in an arginine-to-histidine substitution at codon 537 (p.Arg537His). It was identified in all affected individuals, including the proband, her mother (III.2), aunt (III.3), and cousin (IV.5), all of whom had documented histories of ICP and/or hepatobiliary disease. Interestingly, the variant was also found in two additional female relatives: the proband’s younger sister (IV.3) and the daughter the cousin IV.5 (V.1). Although neither of these individuals were evaluated for ICP due to their young age and lack of pregnancy history, V.1 underwent cholecystectomy at the age of 11, suggesting that this variant may be associated with early-onset hepatobiliary manifestations, even outside the context of pregnancy.

The *ABCB5* variant identified in this study is not reported in the ClinVar database and is exceedingly rare in population databases. It is classified as pathogenic by the majority of *in silico* prediction tools. Structural analysis of the ABCB5 protein revealed a high likelihood that the p.Arg537His substitution may disrupt the protein structure. This amino acid change is located within the N-terminal “ABC transporter 1” domain, corresponding to NBS1 in canonical ABC transporter architecture. Although this NBS1 module is degenerate, lacking the conserved Walker A motif required for ATP binding and hydrolysis, and therefore, catalytically inactive, it retains important structural and regulatory roles. Degenerate NBS1 domains contribute to overall protein stability, facilitate nucleotide-binding domain dimerization, and help transmit conformational changes to transmembrane domains [23,34]. As such, missense variants located within these regions can still exert deleterious effects on protein function. Notably, the variant was absent in a control group of unrelated women with uncomplicated pregnancies, further supporting its potential relevance to disease susceptibility.

Given the novelty of this finding, we next explored the known biological roles and disease associations of *ABCB5* to evaluate its potential relevance in the pathophysiology of ICP. ABCB5, a member of the ATP-binding cassette (ABC) transporter superfamily, is involved in the ATP-dependent transmembrane transport of structurally diverse substrates [35]. Its expression has been documented in various tissues, including pigment-producing cells such as melanocytes and retinal pigment epithelial cells, as well as in the testis and mammary tissue, and in all compartments of the liver, including hepatocytes, portal veins, and bile ducts [36,37]. Moreover, *ABCB5* is expressed in various malignancies, including colorectal, hepatic, and breast cancers, leukemia, and squamous cell carcinomas of the head, neck, and oral cavity [35,38,39,40,41]. The ABCB5 transporter has been shown to mediate the efflux of several chemotherapeutic agents, including 5-fluorouracil and doxorubicin, contributing to drug resistance in tumor cells [38,42]. Furthermore, *ABCB5* is among the most frequently mutated genes in melanoma, and its genetic variants have also been linked to hepatocellular carcinoma susceptibility [43,44].

Beyond oncology, *ABCB5* has been implicated in stem cell biology. It serves as a marker of limbal stem cells and has been shown to be essential for their maintenance, as well as for corneal development and epithelial repair, as demonstrated by Ksander et al. [45]. Several GWAS studies have also reported potential associations between *ABCB5* variants and diverse clinical phenotypes. For instance, *ABCB5* variants have been associated with early neurological instability following ischemic stroke [46], while deletions encompassing the *ABCB5* gene have been linked to childhood obesity in African American and European–American children [47]. A study conducted in a Korean cohort suggested that a specific four-allele combination, including the *ABCB5* rs17143187 variant, may be associated with idiopathic recurrent pregnancy loss [48]. In atherosclerosis, *ABCB5* expression was markedly upregulated in high-risk atherosclerotic plaques and localized to macrophages in areas of neovascularization and intraplaque hemorrhage [49]. However, it is important to note that most of these studies establish statistical associations rather than direct mechanistic links. The functional role of *ABCB5* in most of these conditions, including neurological, metabolic, reproductive, and cardiovascular diseases, remains to be fully elucidated.

Taken together, *ABCB5* is primarily known for its functions in drug resistance and stem cell biology, and, to date, no direct experimental evidence links this gene to ICP, cholestasis in general, or bile acid transport. Although *ABCB5* has not been previously implicated in hepatobiliary function, its membership in the ABC transporter superfamily, which includes key regulators of bile acid homeostasis, such as *ABCB4*, *ABCB11*, and *ABCC2*, raises the possibility of its unrecognized role in hepatic physiology. To our knowledge, only one previous study has suggested a potential association between *ABCB5* and ICP. In a recent WES-based investigation in Chinese ICP patients, rare variants in multiple ABC transporter genes were identified, including novel pathogenic variants in *ABCB4*, *ABCB11*, and *ABCC2*. Notably, a functional missense variant in *ABCB5* (p.Ser925Ile) was also reported in one patient [17]. However, this study did not include further functional validation, and the mechanistic involvement of *ABCB5* in ICP pathophysiology remains speculative. Therefore, our findings may represent the first step toward identifying a previously unrecognized link between *ABCB5* and familial ICP. Functional studies, including in vitro bile acid transport assays and animal models, are warranted to investigate the biological role of *ABCB5* in hepatobiliary homeostasis and to determine whether it represents a novel susceptibility gene for ICP.

This study has several limitations that should be considered when interpreting the results. Most importantly, the genetic findings are based on a single family, limiting the generalizability of the observations. Although the *ABCB5* variant segregated with hepatobiliary symptoms in multiple affected individuals, no functional experiments were performed to assess its biological effects directly. DNA was also unavailable from some potentially informative family members, such as the proband’s grandfather, which restricted the ability to fully reconstruct variant inheritance. The use of WES, while effective for detecting coding variants, does not capture non-coding regions, structural rearrangements, or copy number variations that might also influence disease risk. Finally, the *ABCB5* variant was not observed in 433 unrelated control individuals of similar ethnic background, but its frequency and significance in broader ICP populations remain to be established. Future studies in larger, independent cohorts and functional validation will be essential to better define the role of *ABCB5* in cholestatic disorders.

## 5. Conclusions

In conclusion, this study identifies a rare missense variant in the *ABCB5* gene as a potential novel genetic contributor to familial intrahepatic cholestasis of pregnancy. Although *ABCB5* has not previously been implicated in bile acid metabolism or hepatobiliary function, its role as an ABC transporter and its expression in various tissues suggest plausible involvement. The consistent segregation of the *ABCB5* variant with hepatobiliary phenotypes across multiple affected family members underscores its potential pathogenic relevance. These findings expand the spectrum of candidate genes associated with ICP and warrant further studies to elucidate the biological role of *ABCB5* in liver physiology and bile acid homeostasis.

## Data Availability

The de-identified datasets generated through this study can be provided by the corresponding author upon request.

## Supplementary Materials

The following supporting information can be downloaded at https://www.mdpi.com/article/10.3390/jcm14165618/s1. Figure S1: Allele frequency of the ABCB5 p.Arg537His (rs779950110) variant across various populations. Data were retrieved from the Genome Aggregation Database (gnomAD, https://gnomad.broadinstitute.org/, accessed on 2 June 2025) and the UCSC Genome Browser 114 (https://genome-euro.ucsc.edu/ accessed on 2 June 2025). * The allele frequency of rs779950110 in the Polish population is based on the analysis of the control group included in the present study; Figure S2: The detection of the ABCB5 missense variant with whole-exome sequencing (WES). WES identified a likely pathogenic variant in exon 14 of the ABCB5 gene. This heterozygous c.1610G>A transition, resulting in a p.Arg537His substitution, was detected in the proband (IV.1) and five of her female relatives who were available for testing: III.2, III.3, IV.3, IV.4, and V.1; Figure S3: Confirmation analysis of the ABCB5 missense variant. The presence of the heterozygous c.1610G>A variant in exon 14 of the ABCB5 gene, resulting in a p.Arg537His substitution, was confirmed in all six tested family members (III.2, III.3, IV.1—proband, IV.3, IV.4, and V.1) using standard Sanger sequencing and high-resolution melting curve analysis; Table S1: *In silico* analysis of the ABCB5 p.Arg537His (rs779950110) variant.

## Abbreviations

The following abbreviations are used in this manuscript:

ADA	Adaptive Boosting
CADD	Combined Annotation-Dependent Depletion
GWAS	Genome-Wide Association Study
HRM	High-Resolution Melting curve
ICP	Intrahepatic Cholestasis of Pregnancy
IUFD	Intrauterine Fetal Death
NBS1	Nucleotide-Binding Site 1
pLDDT	Predicted Local Distance Difference Test
TBA	Total Bile Acids
TSBA	Total Serum Bile Acid
WES	Whole-Exome Sequencing

## References

[1] Geenes V., Williamson C. (2009). Intrahepatic cholestasis of pregnancy. World J. Gastroenterol..

[2] Dixon P.H., Levine A.P., Cebola I., Chan M.M.Y., Amin A.S., Aich A., Mozere M., Maude H., Mitchell A.L., Zhang J. (2022). GWAS meta-analysis of intrahepatic cholestasis of pregnancy implicates multiple hepatic genes and regulatory elements. Nat. Commun..

[3] Ovadia C., Seed P.T., Sklavounos A., Geenes V., Di Ilio C., Chambers J., Kohari K., Bacq Y., Bozkurt N., Brun-Furrer R. (2019). Association of adverse perinatal outcomes of intrahepatic cholestasis of pregnancy with biochemical markers: Results of aggregate and individual patient data meta-analyses. Lancet.

[4] Mitchell A.L., Ovadia C., Syngelaki A., Souretis K., Martineau M., Girling J., Vasavan T., Fan H.M., Seed P.T., Chambers J. (2021). Re-evaluating diagnostic thresholds for intrahepatic cholestasis of pregnancy: Case-control and cohort study. BJOG.

[5] Guszczynska-Losy M., Wirstlein P.K., Wender-Ozegowska E., Kedzia M. (2020). Evaluation of predictive value of biochemical markers for adverse obstetrics outcomes in pregnancies complicated by cholestasis. Ginekol. Pol..

[6] Abu-Hayyeh S., Martinez-Becerra P., Sheikh Abdul Kadir S.H., Selden C., Romero M.R., Rees M., Marschall H.U., Marin J.J., Williamson C. (2010). Inhibition of Na+-taurocholate Co-transporting polypeptide-mediated bile acid transport by cholestatic sulfated progesterone metabolites. J. Biol. Chem..

[7] Abu-Hayyeh S., Papacleovoulou G., Lövgren-Sandblom A., Tahir M., Oduwole O., Jamaludin N.A., Ravat S., Nikolova V., Chambers J., Selden C. (2013). Intrahepatic cholestasis of pregnancy levels of sulfated progesterone metabolites inhibit farnesoid X receptor resulting in a cholestatic phenotype. Hepatology.

[8] Brites D., Rodrigues C.M., van-Zeller H., Brito A., Silva R. (1998). Relevance of serum bile acid profile in the diagnosis of intrahepatic cholestasis of pregnancy in an high incidence area: Portugal. Eur. J. Obstet. Gynecol. Reprod. Biol..

[9] Dixon P.H., Weerasekera N., Linton K.J., Donaldson O., Chambers J., Egginton E., Weaver J., Nelson-Piercy C., de Swiet M., Warnes G. (2000). Heterozygous MDR3 missense mutation associated with intrahepatic cholestasis of pregnancy: Evidence for a defect in protein trafficking. Hum. Mol. Genet..

[10] Stättermayer A.F., Halilbasic E., Wrba F., Ferenci P., Trauner M. (2020). Variants in ABCB4 (MDR3) across the spectrum of cholestatic liver diseases in adults. J. Hepatol..

[11] Pillarisetty L.S., Sharma A. (2025). Pregnancy Intrahepatic Cholestasis. StatPearls [Internet].

[12] Turro E., Astle W.J., Megy K., Gräf S., Greene D., Shamardina O., Allen H.L., Sanchis-Juan A., Frontini M., Thys C. (2020). Whole-genome sequencing of patients with rare diseases in a national health system. Nature.

[13] Meier Y., Zodan T., Lang C., Zimmermann R., Kullak-Ublick G.A., Meier P.J., Stieger B., Pauli-Magnus C. (2008). Increased susceptibility for intrahepatic cholestasis of pregnancy and contraceptive-induced cholestasis in carriers of the 1331T>C polymorphism in the bile salt export pump. World J. Gastroenterol..

[14] Pauli-Magnus C., Meier P.J., Stieger B. (2010). Genetic determinants of drug-induced cholestasis and intrahepatic cholestasis of pregnancy. Semin. Liver Dis..

[15] Tang M., Xiong L., Cai J., Fu J., Liu H., Ye Y., Yang L., Xing S., Yang X. (2024). Intrahepatic cholestasis of pregnancy: Insights into pathogenesis and advances in omics studies. Hepatol. Int..

[16] Liu X., Lai H., Zeng X., Xin S., Nie L., Liang Z., Wu M., Chen Y., Zheng J., Zou Y. (2020). Whole-exome sequencing reveals ANO8 as a genetic risk factor for intrahepatic cholestasis of pregnancy. BMC Pregnancy Childbirth.

[17] Liu X., Lai H., Xin S., Li Z., Zeng X., Nie L., Liang Z., Wu M., Zheng J., Zou Y. (2021). Whole-exome sequencing identifies novel mutations in ABC transporter genes associated with intrahepatic cholestasis of pregnancy disease: A case-control study. BMC Pregnancy Childbirth.

[18] Liu X., Zheng J., Xin S., Zeng Y., Wu X., Zeng X., Lai H., Zou Y. (2022). Whole-exome sequencing expands the roles of novel mutations of organic anion transporting polypeptide, ATP-binding cassette transporter, and receptor genes in intrahepatic cholestasis of pregnancy. Front. Genet..

[19] Girling J., Knight C.L., Chappell L., Royal College of Obstetricians and Gynaecologists (2022). Intrahepatic cholestasis of pregnancy: Green-top Guideline No. 43 June 2022. BJOG.

[20] Grochowalski Ł., Jarczak J., Urbanowicz M., Słomka M., Szargut M., Borówka P., Sobalska-Kwapis M., Marciniak B., Ossowski A., Lorkiewicz W. (2020). Y-Chromosome Genetic Analysis of Modern Polish Population. Front. Genet..

[21] Biedziak B., Dąbrowska J., Szponar-Żurowska A., Bukowska-Olech E., Jamsheer A., Mojs E., Mulle J., Płoski R., Mostowska A. (2023). Identification of a new familial case of 3q29 deletion syndrome associated with cleft lip and palate via whole-exome sequencing. Am. J. Med. Genet. Part A.

[22] Jumper J., Evans R., Pritzel A., Green T., Figurnov M., Ronneberger O., Tunyasuvunakool K., Bates R., Žídek A., Potapenko A. (2021). Highly accurate protein structure prediction with AlphaFold. Nature.

[23] Stockner T., Gradisch R., Schmitt L. (2020). The role of the degenerate nucleotide binding site in type I ABC exporters. FEBS Lett..

[24] Gerard L., Gillet J.P. (2024). The uniqueness of ABCB5 as a full transporter ABCB5FL and a half-transporter-like ABCB5β. Cancer Drug Resist..

[25] Dixon P.H., Williamson C. (2016). The pathophysiology of intrahepatic cholestasis of pregnancy. Clin. Res. Hepatol. Gastroenterol..

[26] Mikucka-Niczyporuk A., Pierzynski P., Lemancewicz A., Kosinski P., Charkiewicz K., Knas M., Kacerovsky M., Blachnio-Zabielska A., Laudanski P. (2020). Role of sphingolipids in the pathogenesis of intrahepatic cholestasis. Prostaglandins Other Lipid Mediat..

[27] Li J., Chen J., Lee P.M.Y., Zhang J., Li F., Ren T. (2023). Familial clustering of intrahepatic cholestasis of pregnancy: A nationwide population-based study in Denmark. Hepatology.

[28] Turunen K., Helander K., Mattila K.J., Sumanen M. (2013). Intrahepatic cholestasis of pregnancy is common among patients’ first-degree relatives. Acta Obstet. Gynecol. Scand..

[29] Davit-Spraul A., Gonzales E., Baussan C., Jacquemin E. (2009). Progressive familial intrahepatic cholestasis. Orphanet J. Rare Dis..

[30] Pasmant E., Goussard P., Baranes L., Laurendeau I., Quentin S., Ponsot P., Consigny Y., Farges O., Condat B., Vidaud D. (2012). First description of ABCB4 gene deletions in familial low phospholipid-associated cholelithiasis and oral contraceptives-induced cholestasis. Eur. J. Hum. Genet..

[31] Burdick K.J., Cogan J.D., Rives L.C., Robertson A.K., Koziura M.E., Brokamp E., Duncan L., Hannig V., Pfotenhauer J., Vanzo R. (2020). Limitations of exome sequencing in detecting rare and undiagnosed diseases. Am. J. Med. Genet. Part A.

[32] Corominas J., Smeekens S.P., Nelen M.R., Yntema H.G., Kamsteeg E.J., Pfundt R., Gilissen C. (2022). Clinical exome sequencing-Mistakes and caveats. Hum. Mutat..

[33] Ewans L.J., Minoche A.E., Schofield D., Shrestha R., Puttick C., Zhu Y., Drew A., Gayevskiy V., Elakis G., Walsh C. (2022). Whole exome and genome sequencing in mendelian disorders: A diagnostic and health economic analysis. Eur. J. Hum. Genet..

[34] Díaz-Anaya A.M., Gerard L., Albert M., Gaussin J.F., Boonen M., Gillet J.P. (2023). The β Isoform of Human ATP-Binding Cassette B5 Transporter, ABCB5β, Localizes to the Endoplasmic Reticulum. Int. J. Mol. Sci..

[35] Chen K.G., Szakács G., Annereau J.P., Rouzaud F., Liang X.J., Valencia J.C., Nagineni C.N., Hooks J.J., Hearing V.J., Gottesman M.M. (2005). Principal expression of two mRNA isoforms (ABCB 5alpha and ABCB 5beta ) of the ATP-binding cassette transporter gene ABCB 5 in melanoma cells and melanocytes. Pigment. Cell Res..

[36] Moitra K., Scally M., McGee K., Lancaster G., Gold B., Dean M. (2011). Molecular evolutionary analysis of ABCB5: The ancestral gene is a full transporter with potentially deleterious single nucleotide polymorphisms. PLoS ONE.

[37] Saeed M.E.M., Boulos J.C., Machel K., Andabili N., Marouni T., Roth W., Efferth T. (2022). Expression of the Stem Cell Marker ABCB5 in Normal and Tumor Tissues. In Vivo.

[38] Frank N.Y., Margaryan A., Huang Y., Schatton T., Waaga-Gasser A.M., Gasser M., Sayegh M.H., Sadee W., Frank M.H. (2005). ABCB5-mediated doxorubicin transport and chemoresistance in human malignant melanoma. Cancer Res..

[39] Frank N.Y., Frank M.H. (2009). ABCB5 gene amplification in human leukemia cells. Leuk. Res..

[40] Grimm M., Krimmel M., Polligkeit J., Alexander D., Munz A., Kluba S., Keutel C., Hoffmann J., Reinert S., Hoefert S. (2012). ABCB5 expression and cancer stem cell hypothesis in oral squamous cell carcinoma. Eur. J. Cancer.

[41] Guo Q., Grimmig T., Gonzalez G., Giobbie-Hurder A., Berg G., Carr N., Wilson B.J., Banerjee P., Ma J., Gold J.S. (2018). ATP-binding cassette member B5 (ABCB5) promotes tumor cell invasiveness in human colorectal cancer. J. Biol. Chem..

[42] Kugimiya N., Nishimoto A., Hosoyama T., Ueno K., Enoki T., Li T.S., Hamano K. (2015). The c-MYC-ABCB5 axis plays a pivotal role in 5-fluorouracil resistance in human colon cancer cells. J. Cell. Mol. Med..

[43] Leung I.C., Chong C.C., Cheung T.T., Yeung P.C., Ng K.K., Lai P.B., Chan S.L., Chan A.W., Tang P.M., Cheung S.T. (2020). Genetic variation in ABCB5 associates with risk of hepatocellular carcinoma. J. Cell. Mol. Med..

[44] Duvivier L., Gillet J.P. (2022). Deciphering the roles of ABCB5 in normal and cancer cells. Trends Cancer.

[45] Ksander B.R., Kolovou P.E., Wilson B.J., Saab K.R., Guo Q., Ma J., McGuire S.P., Gregory M.S., Vincent W.J., Perez V.L. (2014). ABCB5 is a limbal stem cell gene required for corneal development and repair. Nature.

[46] Ibanez L., Heitsch L., Carrera C., Farias F.H.G., Del Aguila J.L., Dhar R., Budde J., Bergmann K., Bradley J., Harari O. (2022). Multi-ancestry GWAS reveals excitotoxicity associated with outcome after ischaemic stroke. Brain.

[47] Glessner J.T., Bradfield J.P., Wang K., Takahashi N., Zhang H., Sleiman P.M., Mentch F.D., Kim C.E., Hou C., Thomas K.A. (2010). A genome-wide study reveals copy number variants exclusive to childhood obesity cases. Am. J. Hum. Genet..

[48] Lee H.A., Ahn E.H., Kim J.H., Kim J.O., Ryu C.S., Lee J.Y., Cho S.H., Lee W.S., Kim N.K. (2018). Association study of frameshift and splice variant polymorphisms with risk of idiopathic recurrent pregnancy loss. Mol. Med. Rep..

[49] Wadén K., Karlöf E., Narayanan S., Lengquist M., Hansson G.K., Hedin U., Roy J., Matic L. (2022). Clinical risk scores for stroke correlate with molecular signatures of vulnerability in symptomatic carotid patients. iScience.

[50] Sawicka-Gutaj N., Gruszczyński D., Guzik P., Mostowska A., Walkowiak J. (2022). Ethics of Human Studies in the Light of the Declaration of Helsinki—A Mini-Review. J. Med. Sci..

