# Supplementary material for: A Rare ABCB5 Variant in a Familial Case of Intrahepatic Cholestasis of Pregnancy: A Potential Novel Genetic Contributor
Source: J Clin Med. 2025 Aug 8;14(16):5618. doi: 10.3390/jcm14165618 (PMC12386353; doi:10.3390/jcm14165618)
Supplement: Supplementary file 1 [file jcm-14-05618-s001.zip › jcm-3741341-Supplementary Table S1.pdf]

Supplementary Table S1. ***In silico*** **analysis of the ABCB5 p.Arg537His (rs779950110) variant**

**Meta scores**			
**Engine***	**Score**	**Calibrated Prediction**	**Version**
**MetaRNN**	0.9794	**Pathogenic Strong**	dbNSFP version 4.9
**MetaLR**	0.9567	**Pathogenic Moderate**	dbNSFP version 4.9
**MetaSVM**	1.0403	**Pathogenic Moderate**	dbNSFP version 4.9
**REVEL**	0.85	**Pathogenic Moderate**	dbNSFP version 4.9
**BayesDel noAF**	noAF score 0.2605	**Pathogenic Supporting**	dbNSFP version 4.9
**BayesDel addAF**	addAF score 0.1268	**Uncertain**	dbNSFP version 4.9
**Individual Predictions**			
**Engine***	**Score**	**Calibrated Prediction**	**Version**
**MutPred**	0.973	**Pathogenic Strong**	dbNSFP version 4.9
**PolyPhen-2**	0.999	**Probably Damaging**	version 2
**LIST-S2**	0.9827	**Pathogenic Moderate**	dbNSFP version 4.9
**Mutation assessor**	5.12	**Pathogenic Moderate**	dbNSFP version 4.9
**EIGEN**	raw coding 0.7821	**Pathogenic Supporting**	dbNSFP version 4.9
**EIGEN PC**	PC raw coding score 0.6336	**Pathogenic Supporting**	dbNSFP version 4.9
**MVP**	0.95	**Pathogenic Supporting**	dbNSFP version 4.9
**PROVEAN**	-4.58	**Pathogenic Supporting**	dbNSFP version 4.9
**SIFT**	0	**Pathogenic Supporting**	dbNSFP version 4.9
**SIFT4G**	0	**Pathogenic Supporting**	dbNSFP version 4.9
**AlphaMissense**	0.3009	**Benign Supporting**	version 03-Jul-2024
**LRT**	0.003304	**Benign Supporting**	dbNSFP version 4.9
**MutationTaster**	0.9973	**Benign Supporting**	dbNSFP version 4.9
**BLOSUM**	-1	**Uncertain**	version BLOSUM100
**DANN**	0.9992	**Uncertain**	dbNSFP version 4.9
**DEOGEN2**	0.6804	**Uncertain**	dbNSFP version 4.9
**FATHMM**	-3.72	**Uncertain**	dbNSFP version 4.9
**FATHMM-MKL**	coding score 0.9497	**Uncertain**	dbNSFP version 4.9
**FATHMM-XF**	coding score 0.6903	**Uncertain**	dbNSFP version 4.9
**M-CAP**	0.178	**Uncertain**	dbNSFP version 4.9
**PrimateAI**	0.6499	**Uncertain**	dbNSFP version 4.9

*Results were retrieved from the human genomic variant search engine VarSome1 (https://varsome.com/) and the Ensembl genome browser (https://www.ensembl.org/index.html).
