# Supplementary figures and images for: A Rare ABCB5 Variant in a Familial Case of Intrahepatic Cholestasis of Pregnancy: A Potential Novel Genetic Contributor
Source: J Clin Med. 2025 Aug 8;14(16):5618. doi: 10.3390/jcm14165618 (PMC12386353; doi:10.3390/jcm14165618)

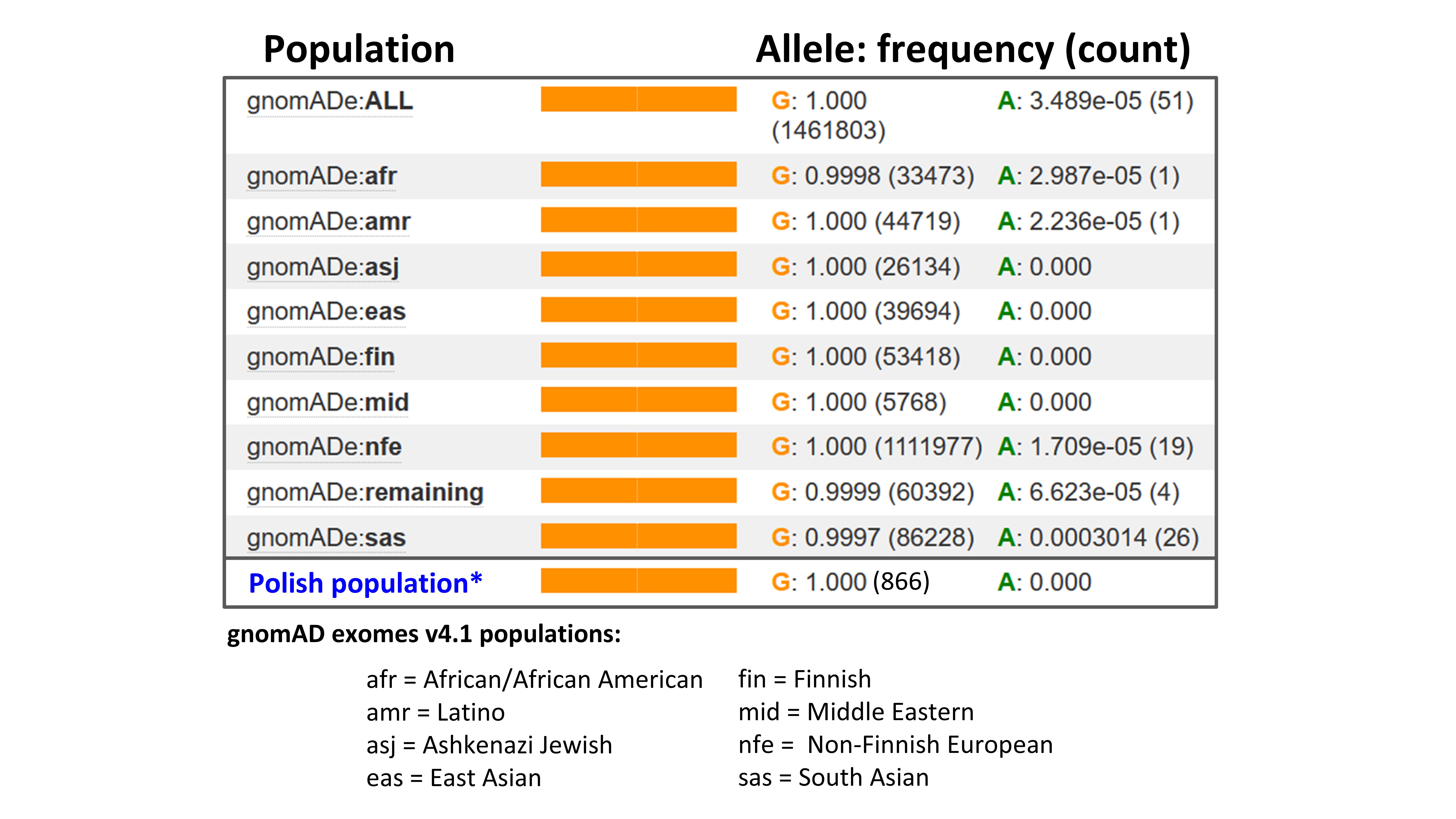

Supplement: Supplementary file 1 [file jcm-14-05618-s001.zip › jcm-3741341-Supplementary Figure S1.jpg]

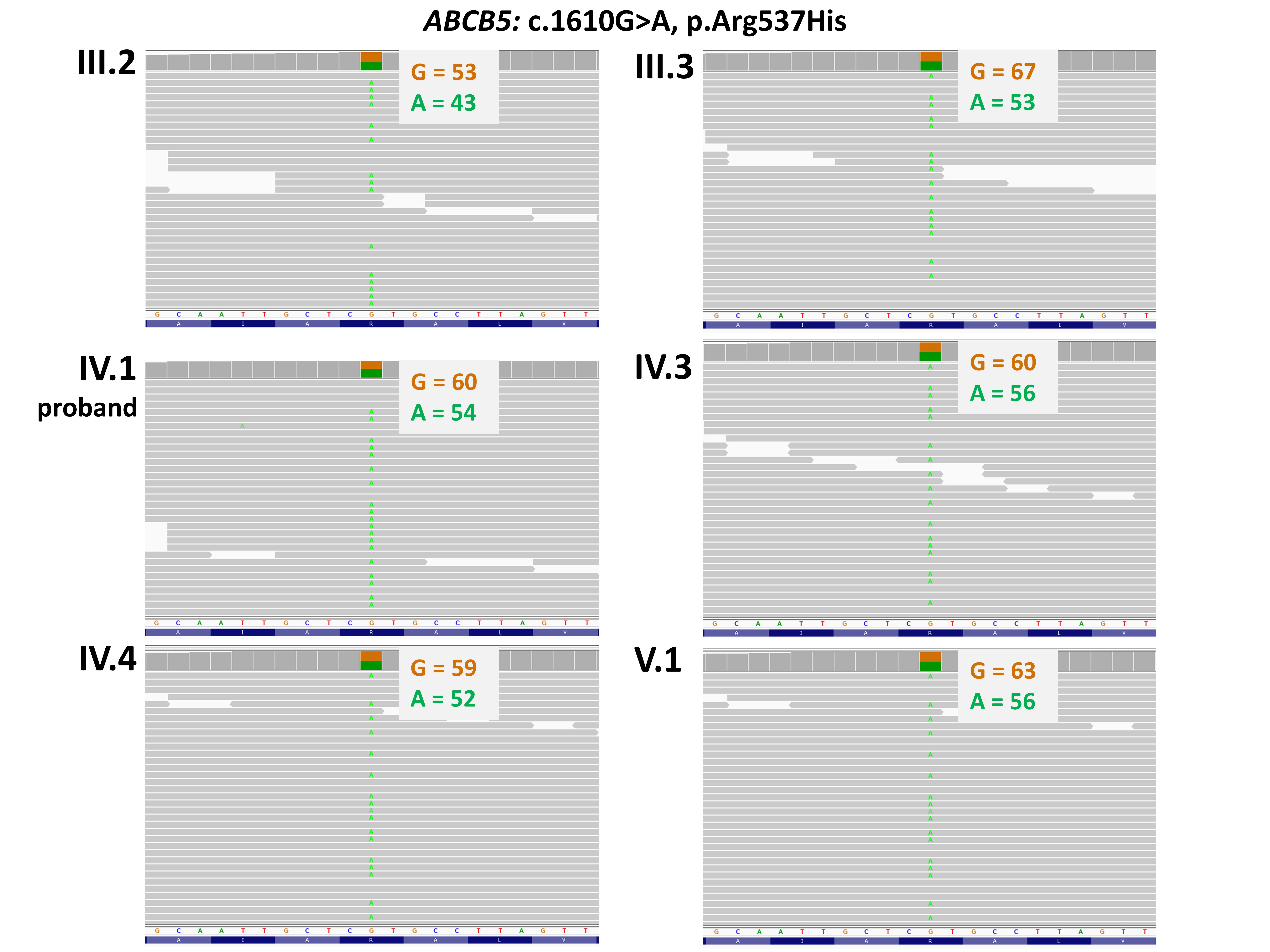

Supplement: Supplementary file 1 [file jcm-14-05618-s001.zip › jcm-3741341-Supplementary Figure S2.jpg]

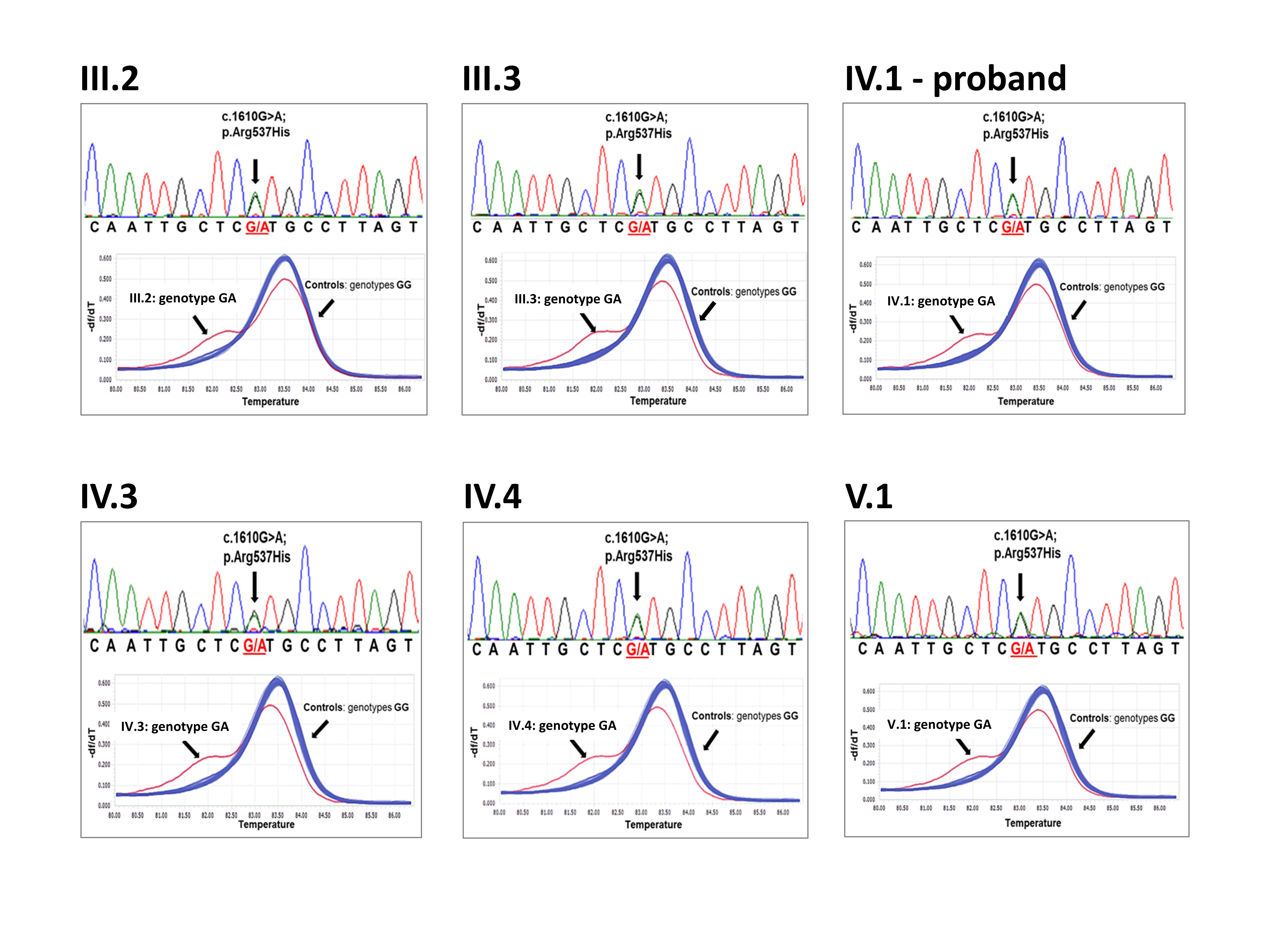

Supplement: Supplementary file 1 [file jcm-14-05618-s001.zip › jcm-3741341-Supplementary Figure S3.jpg]
